# New candidate therapeutic agents for endometrial cancer: Potential for clinical practice (Review)

**DOI:** 10.3892/or.2013.2221

**Published:** 2013-01-03

**Authors:** KIYOKO UMENE, KOUJI BANNO, IORI KISU, MEGUMI YANOKURA, YUYA NOGAMI, KOSUKE TSUJI, KENTA MASUDA, ARISA UEKI, YUSUKE KOBAYASHI, WATARU YAMAGAMI, EIICHIRO TOMINAGA, NOBUYUKI SUSUMU, DAISUKE AOKI

**Affiliations:** Department of Obstetrics and Gynecology, School of Medicine, Keio University, Tokyo 160-8582, Japan

**Keywords:** endometrial cancer, fourth-generation progestins, metformin, microRNA, mTOR inhibitors

## Abstract

Cases of endometrial cancer have increased in recent years, but the prognosis of patients with this disease has also been improved by combined modality therapy with surgery, radiotherapy and chemotherapy. However, the development of new therapy is required from the perspectives of conservation of fertility and efficacy for recurrent and intractable cancer. New candidate therapeutic agents for endometrial cancer include fourth-generation progestins for inhibition of growth and differentiation of endometrial glands; metformin for reduction of hTERT expression in the endometrium and inhibition of the mTOR pathway by activation of AMPK, with consequent inhibition of the cell cycle; mTOR inhibitors for supressing growth of cancer cells by G1 cell cycle arrest; microRNAs involved in the molecular mechanisms of oncogenesis and progression; and HDAC inhibitors that block the growth of cancer cells by transcriptional elevation of tumor-suppressor genes, cell cycle arrest and induction of apoptosis. In this study, we review the background and early clinical evidence for these agents as new therapeutic candidates for endometrial cancer.

## 1. Introduction

Endometrial cancer is a malignant epithelial tumor that occurs in the endometrium and frequently develops in perimenopausal women aged 50–60 years. Major symptoms include dysfunctional uterine bleeding, hypermenorrhea, irregular menstruation and sterility. The prevalence and mortality of endometrial cancer in Japan have increased: in 1985 and 2007, 2,636 and 9,104 women were diagnosed with the disease, and 522 and 1,637 deaths were reported due to the disease, respectively (1). These values are not age-dependent, but are likely to increase further in Japan due in part to the aging population. The recent increase in the cases of endometrial cancer is associated with changes in the lifestyle of women, including western dietary habits, delayed marriage and decreased gravidity (2). Endometrial cancer is classified into a type I, which is estrogen-dependent, and a type II estrogen-independent form. The type I accounts for approximately 80% of all cases of endometrial cancer. Risk factors include late menopause, nulliparity, obesity and estrogen-producing tumors.

Current therapy for type I endometrial cancer involves the use of surgery, radiotherapy and chemotherapy in combination. This approach has improved the prognosis of patients. Approximately 90% of cases of endometrial cancer are early cancers localized in the uterus (stages I and II) and surgery is the most common therapy; however, this is a problem for young nulliparous women since the uterus is removed, resulting in loss of fertility (3). In addition, advanced cancer is increasing with the absolute increase in patients, and some early cancers recur and metastasize to other organs (i.e., out of the pelvis). Surgery is not applicable in these patients and radiotherapy and chemotherapy are administered. However, the efficacy is limited and the prognosis is poor, indicating the need for new therapy (4). Improved understanding of the mechanisms of oncogenesis and progression are needed for development of this new therapy.

Non-endometrioid adenocarcinoma, the main cancer in the endometrium, includes serous adenocarcinoma, clear cell adenocarcinoma and mucinous adenocarcinoma. Endometrioid adenocarcinoma is distinguished from non-endometrioid adenocarcinoma based on the molecular pathology and clinicopathology, which is not estrogen-dependent and is thought to be a particular type occurring in postmenopausal aged women. The prognosis is poorer due to the presence of poorly differentiated and muscle-invasive adenocarcinoma (5). Cases of non-endometrioid adenocarcinoma also frequently have lesions in regions other than the uterus; therefore, staging laparotomy including pelvic and paraaortic lymph node dissection and omentectomy is needed. Since the presence and size of residual lesions may be important prognostic indicators, the first step of treatment is surgery to reduce the lesions as much as possible. There is no difference in prognosis between chemotherapy and radiotherapy for the whole abdomen as postoperative adjuvant therapy for stage III and IV serous adenocarcinoma (6). In chemotherapy for stage I serous adenocarcinoma, regimens including platinum-based agents are efficacious (7) and combination therapy with taxanes and platinum-based agents demonstrates a high response rate (8). However, the age at onset of non-endometrioid adenocarcinoma patients is higher than that of endometrioid adenocarcinoma patients and the prognosis is poor. Therefore, development of therapy for this type of endometrial cancer is required.

## 2. Fourth-generation progestins

Type I endometrial cancer is an estrogen-dependent cancer. Gestagens (progestogens) are estrogen antagonists that were invented to treat progression of symptoms caused by estrogen. Progestin is a synthetic gestagen with progesterone action and androgenicity that inhibits growth and differentiation of endometrial glandular epithelial cells, thereby reducing endometriosis and endometrial cancer. Progestin decreases expression of the estrogen receptor (ER) (9), represses ER-related transcription of genes involved in cell growth (10), and activates the tumor-suppressor gene *p21*(11) as mechanisms of inhibition of proliferation of endometrial cancer cells. Hormone therapy with progestin is conducted only for atypical endometrial hyperplasia and endometrial cancer of Ia, the patient with advanced endometrial cancer who cannot be treated with anticancer agents, and the patient who wants to maintain fertility. However, total hysterectomy is superior to hormone therapy in terms of safety and cure rate.

**Figure 1:**
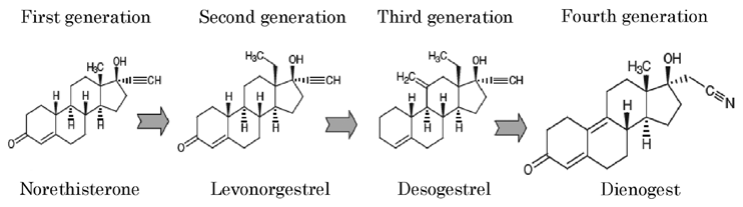
Structures of different generations of progestins.

Progestins are classified into first- to fourth-generation agents. Typical first-, second- and third-generation progestins used clinically include norethisterone, levonorgestrel and desogestrel, respectively. A newer molecule, dienogest, is classified as a fourth-generation agent (Fig. 1). The first-generation progestins have progesterone action and androgenicity, the second generation has more potent progesterone action and relatively greater androgenicity and the third generation has even more potent progesterone action and relatively less androgenicity. However, adverse reactions including acne, hirsutism, obesity, increased libido and virilism still occur due to remaining androgenicity. Thus, the fourth-generation progestins were developed as new agents without androgenicity. These progestins are selective progesterone receptor (PR) agonists that inhibit ovulation, follicle development, growth of endometrial cells and cytokine production, which leads to inhibition of estrogen production and efficacy for treatment of estrogen-dependent endometrial cancer.

The fourth-generation progestins also inhibit the growth of endometrial cancer cells that are unresponsive to older progestins such as medroxyprogesterone acetate (MPA). The mechanism of the antitumor effect of the fourth-generation agents is thought to differ from that of earlier progestins (12). Katsuki *et al* investigated the effect of dienogest, a fourth generation progestin, in two human endometrial cancer cell lines: HEC-88nu (ER^+^, PR^-^) and Ishikawa (ER^+^, PR^+^). HEC-88nu cells did not respond to MPA, while dienogest demonstrated growth inhibition of these cells. Both dienogest and MPA inhibited the growth of Ishikawa cells, while dienogest had a similar effect to MPA at doses of 1/100 to 1/10,000 that of MPA. The sensitivity of endometrial cells to MPA is related to the expression of PR; however, dienogest had effects on HEC-88nu cells which do not express PR, suggesting the possibility of antitumor effects on cancers unresponsive to earlier progestins (13).

Inhibition of neovascularization has been proposed as the mechanism underlying the antitumor effect of dienogest. Nakamura *et al* suggested that dienogest blocks neovascularization and inhibits angiogenesis, both of which play important roles in growth, invasion and metastasis of cancer cells. The mechanism of inhibition of neovascularization by dienogest is unclear (14), while Katayama *et al*(15) found that a decrease in smooth muscle α-actin around endometrial vessels caused by dienogest changed the microvascular structure, thereby inhibiting neovascularization in the endometrium. The fourth-generation progestins also do not cause adverse reactions such as decreased bone mineral density, in contrast to GnRH analogs, the first options for endometriosis (16). These progestins are antiandrogenic, but have no androgenic effects such as acne, hirsutism, obesity, increased libido and virilism. Furthermore, the fourth-generation progestins have no steroid hormone action other than progesterone and antiandrogenic activity, and can be used as monotherapy (17). Therefore, these new progestins are likely to be used as the first option for endometriosis based on their safety and potential for long-term use.

Dienogest is a 19-norprogestin with combined properties of 19-norprogestin and progesterone derivatives and has potent progesterone action in the endometrium. Progestine, a common 19-nortestosterone derivative, has a 17 α-ethinyl group in the steroid structure, whereas dienogest has a 17 α-cyanomethyl group and a double bond in the B ring. This produces the specific properties of dienogest described above.

## 3. Metformin

Hyperinsulinemia carries an increased risk for endometrial cancer. In obese patients, excessive insulin is secreted to inhibit hyperglycemia due to insulin resistance; however, insulin itself promotes the growth of cancer cells through stimulation of the activity of insulin-like growth factor-1 (IGF-1). Biguanides improve insulin resistance and decrease the blood insulin concentration, and therefore the efficacy of these drugs for prevention of cancer has been evaluated. Metformin, a biguanide that is commonly used as the first option for type 2 diabetes, has been shown to kill breast cancer stem cells and inhibit cell growth (18) and to inhibit oncogenesis and cell growth in glioma, colon and ovarian cancer. The mechanism of action of metformin involves phosphorylation of LKB-1, which activates AMP-activated protein kinase (AMPK) and consequently inhibits the mammalian target of rapamycin (mTOR) pathway, leading to growth inhibition of cancer cells. Metformin also inhibits the actions of human epidermal growth factor receptor type 2 (HER2), which is directly involved in the growth, metastasis and malignant progression of breast cancer, and aromatase, an enzyme producing estrogen, and improves insulin resistance (19).

Metformin enhances PR expression in the endometrium by inhibition of IGF-1 and IGF-2. This action is also caused by activation of AMPK and inhibition of the mx+TOR signaling pathway, leading to reduced invasion and metastasis of endometrial cancer (20). Metformin also inhibits the cell cycle by reducing the expression of hTERT. Metformin administered with medroxyprogesterone acetate antagonizes IGF-2 and enhances PR expression, providing an effective combination therapy (21). Therefore, metformin has potential as a new therapeutic agent for the prevention and treatment of endometrial cancer. Furthermore, metformin inhibits the replication competence and signaling of cancer stem cells by regulating the expression of microRNAs through a mechanism that remains unclear (22). The potential to target cancer stem cells in various types of cancer may allow metformin to be used in radical cancer treatment.

## 4. mTOR inhibitors

**Figure 2:**
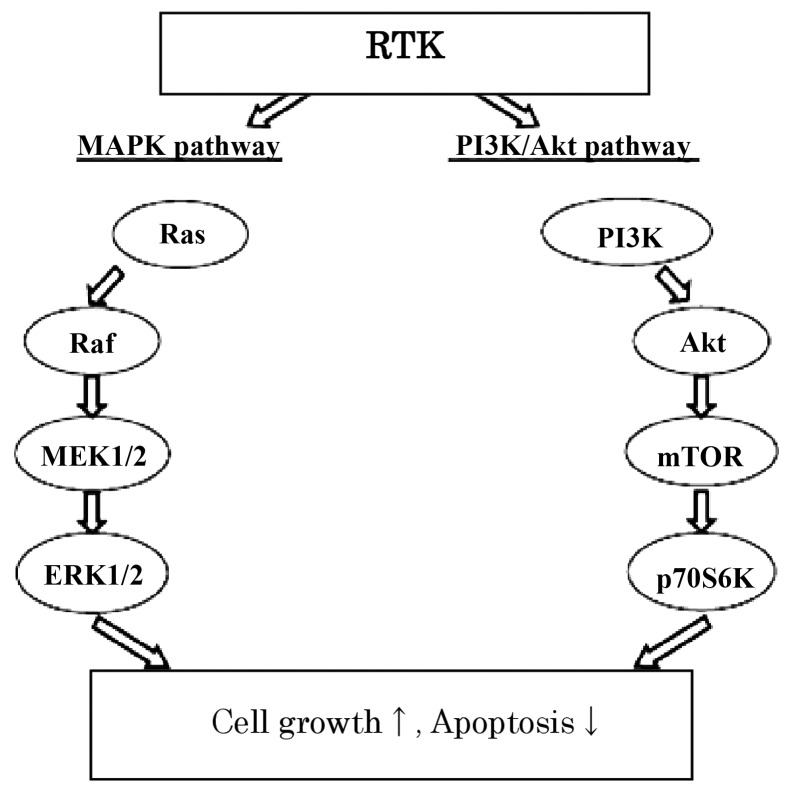
Signaling involved in endometrial oncogenesis.

The signaling network in cells is complicated, and inhibition of one target alone may not have an anticancer effect. Agents with multiple targets are likely to be more effective, and this has led to development of molecular-targeted drugs for endometrial cancer. In endometrial cancer cells, the RAS-RAF and PI3K-Akt-mTOR pathways are activated via receptor tyrosine kinases, including involvement of *PTEN* in the PI3K-Akt-mTOR pathway (Fig. 2). The Wnt signaling pathway, which involves E-cadherin and β-catenin, is also thought to play an important role in the development and malignant progression of endometrial cancer. Therefore, EGF and HER2 signaling inhibitors, angiogenesis inhibitors and molecular-targeted drugs including mTOR inhibitors are used for endometrial cancer. *PTEN* mutations and methylation are common in type I endometrial cancer, which suggests the potential value of treatment with an mTOR inhibitor that blocks the PI3K-AKT-mTOR pathway. *PTEN*, which has an abnormality in 30–50% of cases of endometrial cancer, controls PI3K and inhibits AKT phosphorylation, leading to apoptosis induction. AKT is activated by *PTEN* and cell growth is enhanced via mTOR. mTOR inhibitors block the growth of cancer cells by arresting the cell cycle in the G1 phase (23,24).

mTOR consists of mTOR complexes 1 and 2. The best known mTOR inhibitor, rapamycin, binds to FK506-binding protein-12 to form a complex that inhibits mTOR complex 1. Second-generation mTOR inhibitors inhibit both mTOR complexes 1 and 2, and PI3K-mTOR inhibitors that inhibit both PI3K and mTOR are currently under development (25). Clinical trials of newly developed mTOR inhibitors are ongoing. Ridaforolimus approximately doubled the progression-free survival (PFS) of patients with advanced endometrial cancer compared with conventional endocrine therapy and chemotherapy, and decreased the risk of disease progression by 47%. Thirteen (28%) of 45 patients achieved a clinical beneficial response (CBR), including complete response (CR), partial response (PR) and stable disease (SD), for at least 16 weeks. Ridaforolimus is also an oral drug that is easy to deliver. However, combination of an mTOR inhibitor with endocrine therapy has been shown to increase venous thromboembolism (26). In a phase II study of everolimus, the first oral mTOR inhibitor for endometrial cancer, SD for at least 8 weeks was found in 43% of patients (27). In a phase II study of temsirolimus as first-line treatment in patients with recurrent endometrial cancer who underwent no chemotherapy, 5 (26%) of 19 patients had PR and 12 (63%) had SD (28). A phase II study of temsirolimus at a dose of 25 mg/week for 4 weeks was conducted as second-line treatment in patients with recurrent/advanced endometrial cancer, with the findings of PR in 7.7% and SD in 44.4% (29). These results show that temsirolimus is effective in patients who are unresponsive to chemotherapy.

## 5. microRNAs

Changes in genes involved in oncogenic transformation of endometrial cancer have been observed, but many of the oncogenic mechanisms are not completely understood. Epigenetic mechanisms have attracted attention, and new therapeutic agents for epigenetic regulation at the chromatin level are under development. Hypermethylation of *APC*, *CHFR*, *Sprouty 2*, *RASSF1A*, *GPR54*, *CDH1* and *RSK4* DNA and aberrant methylation of the mismatch repair gene *hMLH1* in the endometrium are thought to be involved in the development of endometrial cancer (30).

Regulation of gene expression by microRNAs is strongly associated with DNA methylation. A microRNA is a short ribonucleic acid (RNA) molecule of 21–25 bases that does not encode a protein. Almost 1,000 microRNAs have been identified in humans. microRNAs are involved in the molecular mechanisms of oncogenesis and progression through inhibitory actions on target molecules. Expression of tumor-suppressor microRNAs is inhibited by epigenomic aberrations, including DNA hypermethylation in cancer cells. Administration of a tumor-suppressor microRNA may recover the function and supplement the activity of the endogenous microRNA and thus may be useful as a cancer therapy (31).

Expression of microRNAs has been examined in endometrial cancer tissues in various phases and stages of differentiation and in normal endometrial tissues. Thirteen microRNAs with significantly differential expression in endometrial cancer tissues and normal tissues have been identified. Eight of the 13 microRNAs showed increased expression and 5 had decreased expression in endometrial cancer (Table I) (32). Functional screening for a cell growth inhibitory effect was conducted using 327 microRNAs to identify tumor-suppressor microRNAs in endometrial cancer. A combination of expression profiling and DNA methylation analysis in endometrial cancer cell lines and tissue specimens identified *miR-152* as a new tumor suppressor by focusing on microRNAs that are frequently inhibited by DNA hypermethylation in endometrial cancer. *miR-152* is thought to play an important role in oncogenesis of endometrial cancer*. E2F3*, *MET* and *Rictor* are target genes of *miR-152*. Tumorigenesis of endometrial cancer cells subcutaneously transplanted in mice was inhibited by administration of *miR-152*, showing the potential for drug discovery based on microRNAs (33). However, the mechanism of tumor suppression is unclear and currently microRNAs cannot be used for diagnosis or treatment of endometrial cancer. Thus, elucidation of this mechanism is required for the development of therapeutic agents based on microRNA activity.

## 6. Histone deacetylase (HDAC) inhibitors

In addition to methylation, deacetylation is an important epigenetic mechanism that influences growth and differentiation of cancer cells and is a potential target in cancer therapy. Histone deacetylases (HDACs) are a class of enzymes that catalyze deacetylation of lysine residues that are acetylated in histone N-terminal domains. HDAC inhibitors enhance transcription of tumor-suppressor genes, arrest the cell cycle and induce apoptosis, and thus are molecular-targeted drugs that inhibit the growth of cancer cells. The major HDACs are trichostatin A, n-butyrate, apicidin and valproic acid (Table II) (30).

A preliminary clinical trial was conducted to investigate the efficacy of HDAC inhibitors in endometrial cancer. The results showed that HDAC inhibitors including vorinostat and valproic acid had potent effects on the inhibition of cell growth in all endometrial cancer cell lines, with G0/G1 or G2/M arrest found after administration of HDAC inhibitors, leading to a significant increase in apoptosis (34). m-Carboxycinnamic acid bis-hydroxamide (CBHA), a new HDAC inhibitor, has also been shown to have potent inhibitory effects on cell growth in endometrial and ovarian cancer cell lines, while having no effect on normal human endometrial glandular epithelial cells at the same concentration; therefore, CBHA may be used as an anticancer agent with few adverse reactions (35). Similar effects have been found for apicidin, another new HDAC inhibitor (36). Thus, HDAC inhibitors may be effective for treatment of endometrial cancer. It has also been suggested that HDAC inhibitors suppress the replication competence of endometrial cancer side-population cells and are effective against cancer stem cells (37). This may permit radical cancer therapy with less metastasis and recurrence through targeting of cancer stem cells with HDAC inhibitors.

## 7. Conclusion

Fourth-generation progestins, metformin, mTOR inhibitors, microRNAs and HDAC inhibitors are promising candidates for treatment of endometrial cancer. The progestins are used as the first option for endometriosis due to their apparent safety in long-term use for estrogen-dependent cancer. Metformin inhibits the mTOR pathway with activation of AMPK, and consequently this drug has potential for prevention and treatment of endometrial cancer. mTOR inhibitors are molecular-targeted drugs that arrest the cell cycle by inhibiting the PI3K-Akt-mTOR pathway. HDAC inhibitors and microRNAs involved in DNA methylation and deacetylation may permit epigenetic regulation of gene expression, which is directly involved in the molecular mechanism of oncogenesis and progression of endometrial cancer.

Further clinical evidence of the effects of all of these agents is needed, since the prevalence and mortality of endometrial cancer are expected to increase further. Accumulation of data in clinical trials is needed to determine efficacy and adverse reactions and to understand the oncogenic mechanism of endometrial cancer and the mechanism of action of the drugs. New agents may also emerge that permit therapy with conservation of fertility, in addition to improved efficacy and reduced adverse reactions, for patients with recurrent and intractable cancer with a poor prognosis.

## Figures and Tables

**Table I:** microRNA expression in endometrial cancer.

microRNA	Expression in endometrial cancer
*miR-185*	↑
*miR-106a*	↑
*miR-181a*	↑
*miR-210*	↑
*miR-423*	↑
*miR-103*	↑
*miR-107*	↑
*let7c*	↑
*let7i*	↓
*miR-221*	↓
*miR-30c*	↓
*miR-152*	↓
*miR-193*	↓

↑, upregulated, ↓, downregulated.

**Table II:** Major HDAC inhibitors with antitumor effect.

HDAC inhibitor	Molecular formula
TrichostatinA	C_17_H_22_N_2_O_3_
Butyric acid	CH_3_(CH_2_)_2_COOH
Apicidin	C_34_H_49_N_5_O_6_
Valproic acid	C_8_H_16_O_2_
Zolinza	C_14_H_20_N_2_O_3_
Chlamydocin	C_28_H_38_N_4_O_6_
